# Nanocarrier-Based Transdermal Drug Delivery Systems for Dermatological Therapy

**DOI:** 10.3390/pharmaceutics16111384

**Published:** 2024-10-28

**Authors:** Yunxiang Kang, Sunxin Zhang, Guoqi Wang, Ziwei Yan, Guyuan Wu, Lu Tang, Wei Wang

**Affiliations:** 1 NMPA Key Laboratory for Research and Evaluation of Cosmetics, China Pharmaceutical University, Nanjing 211198, China; 2 State Key Laboratory of Natural Medicines, Department of Pharmaceutics, School of Pharmacy, China Pharmaceutical University, Nanjing 211198, China

**Keywords:** dermatological therapy, nanocarrier, skin penetration, transdermal delivery

## Abstract

Dermatoses are among the most prevalent non-fatal conditions worldwide. Given this context, it is imperative to introduce safe and effective dermatological treatments to address the diverse needs and concerns of individuals. Transdermal delivery technology offers a promising alternative compared to traditional administration methods such as oral or injection routes. Therefore, this review focuses on the recent achievements of nanocarrier-based transdermal delivery technology for dermatological therapy, which summarizes diverse delivery strategies to enhance skin penetration using various nanocarriers including vesicular nanocarriers, lipid-based nanocarriers, emulsion-based nanocarriers, and polymeric nanocarrier according to the pathogenesis of common dermatoses. The fundamentals of transdermal delivery including skin physiology structure and routes of penetration are introduced. Moreover, mechanisms to enhance skin penetration due to the utilization of nanocarriers such as skin hydration, system deformability, disruption of the stratum corneum, surface charge, and tunable particle size are outlined as well.

## 1. Introduction

Skin, as the largest organ of the human body, is not only related to the appearance of the body but also assumes many important functional roles. Firstly, skin serves as a protective barrier between the internal and external environments, guarding against harmful substances and pathogens. This barrier function helps to prevent infections and maintain overall health [1]. In addition, skin has many critical functions such as regulating body temperature, sensing stimuli, absorbing external substances, secretion, and excretion [2,3,4]. The skin structure consists of three main layers: the epidermis, the dermis, and the subcutaneous tissue. The skin’s outermost layer is the epidermis, which is mainly composed of keratinocytes and can be divided into five sublayers: stratum corneum (SC), stratum lucidum, stratum granulosum, stratum spinosum, and stratum basale [5]. SC is located at the topmost layer of the epidermis and is composed of dead skin cells, forming a protective barrier against the entry of external substances and the loss of water from the body. This barrier property is particularly important when it comes to the penetration of drugs into the skin because SC impedes their absorption [6]. The dermis lies beneath the epidermis and mainly consists of collagen and elastic fibers. These components provide the skin with its strength and elasticity. The dermis is also home to various structures, including blood vessels, sweat glands, sebaceous glands, and sensory receptors, all of which contribute to the skin’s functions [7]. Lastly, subcutaneous tissue, which can be found in the deepest layer of the skin, comprises fat cells and connective tissues. This layer acts as a cushion, protecting underlying structures, and serves as a thermal insulator, aiding in regulating body temperature. Additionally, it functions as a vital energy store for the body [8]. In conclusion, the skin is a complex and versatile organ that goes beyond its superficial appearance. Its multiple layers and intricate structure allow it to fulfill vital functions. Furthermore, the skin acts as a major obstacle mainly due to the existence of SC, which diminishes the penetration of drugs into the body, making it an important consideration factor in transdermal drug delivery systems.

Dermatoses, as a global public health issue, significantly impact the well-being and livelihood of individuals. Statistics have revealed that approximately 10% of the world’s population is afflicted with various dermatoses including vitiligo, psoriasis, melanoma, and others [9]. These disorders not only cause physical discomfort and distress for the patients but also exert a detrimental effect on their mental and social state. Given this context, it becomes imperative to introduce safer and more effective dermatological treatments to address the diverse needs and concerns of individuals. By doing so, we can alleviate the physical and psychological burden caused by dermatoses and empower people to embrace a healthier lifestyle.

Over the past two decades, there has been significant research and development in the field of transdermal delivery technology. The main goal of these studies is to address the challenge of penetration across the skin barrier for the efficient delivery of drugs. Transdermal delivery technology offers a promising alternative compared to traditional drug administration methods such as oral or injection routes because this administration route shows the potential to enhance the therapeutic effects of medications. Owing to the complexities of skin penetration, researchers have designed innovative strategies and technologies to optimize the delivery of therapeutic agents through the skin, including the development of novel drug formulations, such as nanoemulsions and microencapsulated systems, as well as the use of physical enhancement techniques like iontophoresis and electroporation [10]. These extensive studies conducted in this field have contributed to the advancement of transdermal drug delivery technology, which has the potential to revolutionize the way in which active ingredients in medicines are administered, ultimately benefiting patients.

In this review, the recent achievements in nanocarrier-based transdermal technology are summarized, which focuses on the great potential and advantages of nanocarriers in dermatological therapy. Moreover, different nanocarriers-based transdermal delivery strategies and their penetration mechanisms are emphasized as well. We discuss the composition of various nanocarriers, as well as their advantages and limitations in topical application.

## 2. Skin Penetration of Nanocarrier-Based Transdermal Delivery System

### 2.1. Routes of Penetration

**Figure 1:**
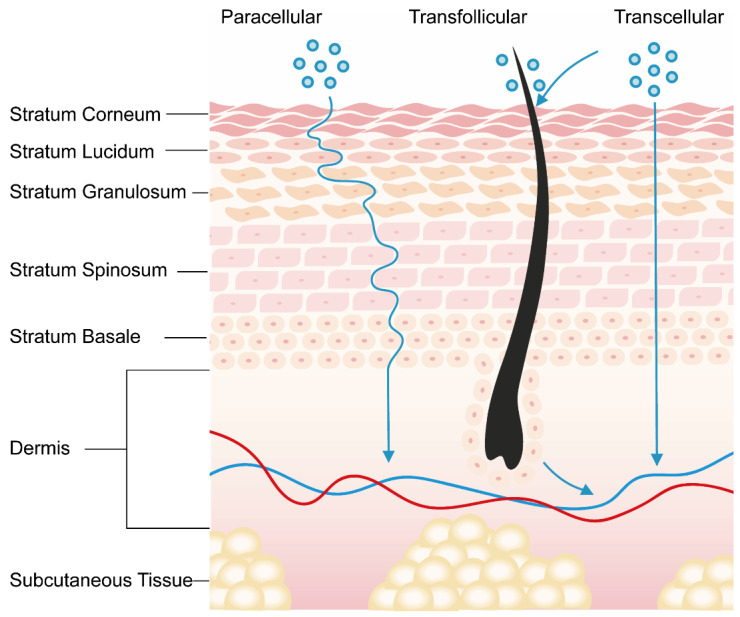
Schematic representation of drug permeation routes across the skin.

Generally, drug molecules are conveyed through the SC via three possible routes (Figure 1): paracellular (intercellular), transcellular (intracellular), and appendageal (transfollicular).

#### 2.1.1. Transcellular Route

In the transcellular pathway, molecules penetrate the matrix (cytoplasm) of dead keratinocytes and the lipids surrounding them, which contain highly hydrated keratin, and drug molecules diffuse alternately in the aqueous and lipid phases. While this is the shortest pathway, molecules need to repeatedly pass through lipophilic cell membranes and aqueous cell contents, which remains challenging for most molecules.

#### 2.1.2. Paracellular Route

The intercellular pathway is that in which drug molecules bypass keratinocytes and penetrate the skin through the interstitium of cells tightly packed between keratinocytes. The tiny channels or gaps that form between keratinocytes are mainly composed of lipids and are more permeable compared to lipid-soluble molecules. Since the molecules do not pass directly through the cells, cell damage can be reduced. However, water-soluble molecules do not readily pass through the intercellular matrix composed of lipids, and the natural barrier functions of the skin (e.g., the tight arrangement of the SC and the presence of a lipid layer) may impede the penetration of certain molecules.

#### 2.1.3. Transfollicular Route

The bypass pathway mainly relies on the penetration of skin appendages such as hair follicles, sebaceous glands, and syringe orifice, and can enter the skin through macromolecular substances and ionic substances that find it difficult to pass through the lipid-rich SC. However, the use of this pathway is somewhat limited compared to the trans epidermal route due to the small absorption area (appendages make up about 0.1% of the total skin surface area) [11].

### 2.2. Mechanism of Enhanced Penetration of Nanocarrier-Based Transdermal Delivery System

#### 2.2.1. Skin Hydration

The barrier function of the skin is mainly maintained by its outermost SC. The hydration of the SC is crucial in regulating barrier function. When the skin is exposed to humidity, keratinocytes absorb large amounts of water. At high relative humidity, hydration also leads to increased SC permeability, which is utilized in transdermal drug delivery and is known as “occlusion” [12]. The penetration of chemicals into the skin is enhanced under occlusive conditions, which is considered to improve skin hydration by preventing trans epidermal water loss and subsequently increasing skin delivery [13].

During transdermal drug delivery, it is important to overcome the skin SC barrier and improve skin permeability. Skin hydration can be positively altered by applying occlusive formulations or imposing occlusive conditions [14]. Increased adhesiveness to surfaces is a general property of ultrafine materials, and the adhesiveness increases with decreased particle size [15]. Therefore, nanocarriers exhibit a distinct adhesiveness to superficial SC. Furthermore, this close contact, referring to the occlusion layer formed on the skin surface, effectively prevents water evaporation from the skin. As water diffuses back into the SC, it leads to hydration, causing the corneocytes to be less packed and have wider gaps between them. Consequently, the barrier function of the SC is compromised, facilitating the delivery of permeants [16]. The lipid nanoparticles adhering to the skin lead to the formation of a film with a subsequent occlusive effect. Elmowafy et al. found that nanostructured lipid carriers (NLCs) can produce an occlusive effect, which may limit the loss of water due to the presence of solid lipids in the NLC lipid matrix [17].

#### 2.2.2. System Deformability

The deformability of drug carriers means that the nanocarriers can change shape or structure when external conditions (e.g., shear, pH, temperature, etc.) change. The deformability of drug carriers can be increased by adding membrane softeners (e.g., deoxycholic acid, etc.) to the prescription so that they can better adapt to the complex environment in vivo and improve drug delivery efficiency and therapeutic efficacy. Deformability is a key property of drug carriers and is commonly used to increase skin penetration without disrupting the SC barrier [18]. Transferosomes show superiority in terms of system flexibility and elasticity. System deformation is induced by edge activators, which have single chains with a high radius of curvature that destabilize phospholipids, forcing amphiphilic molecules to redistribute, leading to increased flexibility of the lipid bilayer [19]. Hydrophilic elastic vesicles are extruded into the narrow intercellular channels of the SC under the influence of a trans-epidermal water activity gradient, which consists of microchannels formed by water-rich zones surrounded by polar lipids, leading to a widened intercellular pathway of 20–30 nm [20]. The deformable transferosomes then move along the transdermal hydration gradient through the microchannels of the skin into the skin layers in response to external pressure within the intercellular lipid accumulation of the SC. Carvalheiro et al. found that the use of a deformable lipid vesicle loaded with Amphotericin B (AmB) delivered directly to the site of action could increase drug retention at the site of action and prolong the local therapeutic effect compared with free AmB [21].

#### 2.2.3. Disruption of the SC

One of the common mechanisms among nanocarriers is disruption of the SC, which is mostly associated with the presence of penetration enhancers. They have the ability to disturb the extremely well-organized structure of the SC, remove and solubilize its lipid and/or keratin contents, and fluidize its crystalline structure [11]. More specifically, the inclusion of ethanol in the bilayers of ethosomes is responsible for their improved skin penetration and bioavailability when compared to traditional vesicular delivery systems [22]. Ethanol interacts with the lipid elements in the polar group area of the SC to modify the structure of the lipophilic or keratinized domains, lower the transition temperature of the lipids, and cause the tightly packed SC lipids to fluidize and break. Therefore, the SC lipids may fluidize and be disturbed by ethanol-based nanocarriers [23]. In addition, studies have shown that nanoemulsions can open up new channels for the penetration of medicinal preparations through the skin accessory structures by changing the SC structure and the spatial conformation of keratin [24].

#### 2.2.4. Surface Charge

Despite the fact that studies have shown that skin penetration may be affected by altering the surface charge of nanocarriers, the kind of charge that results in greater skin penetration is highly debatable in the literature. It is generally known that the skin may function as a negatively charged membrane since the SC lipid layer has a high percentage of negatively charged lipids [25]. Tupal et al. found that neutral nanocarriers appear to penetrate the skin more successfully than charged ones. They claimed that the SC’s net negative charge might either trap positively charged nanoparticles in the upper skin layers before they penetrate deeper levels or stop negatively charged ones from diffusing through the SC [26]. Qu et al. reported that cationic nanomedicines interact with the negative charge of the skin surface via strong electrostatic attractions, leading to a stronger affinity between them. The results showed that the increased skin retention of cationic liposomes could be beneficial for enhancing the results of treatment for chronic dermatoses [27]. Similarly, Wu et al. studied the distribution and penetration of charged nanoparticles in the skin and discovered that in in vitro skin penetration assays, cationic nanoparticles showed a strong affinity for negatively charged skin surfaces [28]. Negatively charged liposomes, on the other hand, have demonstrated their ability to facilitate drug delivery through the skin. Ternullo et al. determined that when deformable liposomes are negatively charged, better retention of human epidermal growth factor on the skin surface is achieved [29]. This behavior could be explained by the repulsive forces between the particles and the skin that form temporary channels in the skin, allowing the particles to penetrate [30].

#### 2.2.5. Particle Size

Particle size influenced the number of drug molecules that pass through the SC. In general, compared to larger particles, smaller particles typically show superior particle/skin adhesion and occlusion [31]. As a result, skin penetration was enhanced. Xiang et al. found that small-sized nanocrystals (NCs) improved passive skin penetration of hesperidin into the SC and deeper skin layers. Compared to 120 nm and 480 nm CUR-NCs, 60 nm NCs possessed greater solubility, which led to larger gradients of concentration at early stages [32]. According to Fick’s first law, this concentration gradient leads to increased passive diffusion through the skin.

## 3. Nanocarriers for Transdermal Delivery

**Figure 2:**
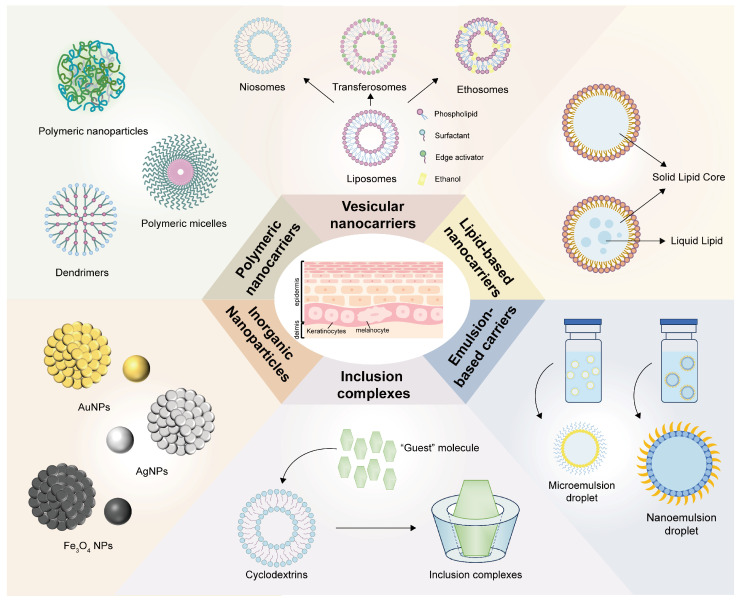
Schematic demonstration of various nanocarriers.

Nanocarriers are widely used in topical applications, including vesicular nanocarriers (liposomes, transferosomes), lipid-based nanocarriers (solid lipid nanoparticles, nanostructured lipid carriers), emulsion-based nanocarriers, polymeric nanocarriers, inorganic nanoparticles, and inclusion complexes [33] (Figure 2). Recently, these have been utilized to deliver various drugs via the transdermal route, as shown in Table 1.

### 3.1. Vesicular Nanocarriers

#### 3.1.1. Liposome

Liposomes, which are classical vesicles composed of phospholipids and cholesterol, were one of the first vesicular carriers studied in this field [64]. They have been widely studied as a vehicle for topical drug delivery since 1968 [65]. When phospholipids are dispersed in water or an aqueous environment, they tend to form spherical vesicles, encapsulating water and soluble solutes in the process. Liposomes have the versatile property of encapsulating hydrophilic and lipophilic drugs. These biodegradable phospholipids are arranged in one or more concentric bilayers with an aqueous core.

Liposomes are suitable for topical transdermal delivery due to their composition, surface properties, and size [34]. When applied to the skin, they adhere to the lipids in the SC, and this adsorption promotes mixing with the SC lipids and achieves the local release of the drug. These vesicles penetrate into the skin via the trans-epidermal or trans-epiphyseal route [66]. In some cases, penetration enhancers are added to the formulation along with liposomes. Penetration enhancers fluidize the SC, which aids in the penetration of liposomes [67].

However, the ability of liposomes to facilitate drug penetration into the deeper layers of the skin is limited, and conventional liposomes have been shown to have minimal ability to enhance drug penetration into deeper regions of the skin and body circulation [68]. In addition, environmental or physical factors can lead to the degradation of liposomes, including aggregation/flocculation and fusion/agglomeration, size, and drug loss [69]. Therefore, researchers have continued to conduct in-depth studies on functionalized modifications of liposome surfaces to overcome the limitations of conventional liposomes themselves [70,71].

#### 3.1.2. Niosome

Niosomes, which were first reported in the early 1970s, are self-assembled vesicles made from nonionic surfactants, cholesterol, and other amphiphilic molecules. Nonionic surfactants are the main element in the preparation of niosomes, amphiphilic molecules with polar heads and nonpolar tails, and this uncharged surfactant has higher stability and less toxicity compared to anionic, cationic, and amphoteric surfactants [72]. In addition, it also possesses the ability to inhibit p-glycoprotein, reduce hemolysis and irritation of cell surfaces, enhance permeability, and improve solubility [73].

Compared to liposomes, niosomes have been introduced as highly effective novel drug carriers due to their advantages such as increased skin penetration, bioavailability, surface adherence, sustained-release qualities, and improved drug stability after capture [35]. Furthermore, niosomes show reduced toxicity, allowing for controlled delivery of loaded drugs [36]. Despite extensive research on niosomes, multiple challenges remain for future clinical applications. The main obstacle that prevents the utilization of niosomes as a potential drug delivery system is sterilization; however, high temperatures tend to destroy their own structure, therefore preparation under sterile conditions is an optional solution [37].

#### 3.1.3. Transferosome

Cevc et al. introduced a new type of carrier system in the 1990s, the transporter, also known as flexible liposomes [74]. Transferosomes are typically composed of phospholipids and edge activators (EAs), which are membrane softeners (e.g., Tween 80, Span 80, and sodium cholate) that are highly deformable.

Transferosomes are highly deformable and hyperflexible vesicles whose penetration into the skin is primarily dependent on the membrane flexibility, hydrophilicity, and maintainable vesicle integrity of transferosomes. In an aqueous environment, phospholipids self-assemble into flexible lipid bilayers and tightly form vesicles [75]. EAs such as membrane instability factors enhance the membrane fluidity and elasticity of transferosomes when binding to lipids in appropriate proportions, which is capable of changing its membrane flexibility and spontaneously passing through the skin pores, resulting in a higher skin permeability [38,39,40]. The permeability gradient is the primary driver of transferosomes into the deeper skin layers [76]. Another possible mechanism of permeation suggests that deformable vesicles act as permeation enhancers and can alter the intercellular lipid composition in the SC, thereby facilitating the transport of the free drug [77]. Therefore, self-optimizing and highly deformable transferosomes have been widely used in the field of transdermal drug delivery and successfully used in a wide range of preclinical trials and various phase I and phase II clinical trials [78].

However, limitations in the use of transferosomes are caused by skin irritation that is attributed to the use of EAs and the purity of phospholipids in the formulation [40]. In addition, transferosomes are susceptible to oxidative degradation by the environment and are chemically unstable. To overcome these stability problems, freeze-drying, optimal storage conditions, degassing, and inert gas purging are mostly used [76].

#### 3.1.4. Ethosome

Ethosomes were first developed by Touitou et al. [79]. Ethosomes are vesicles composed of phospholipids and high concentrations of ethanol (20–45%) and water. Depending on the ethanol content, there are also two subtypes of ethosomes: binary ethosomes and transethosomes. Transethosomes exhibit smaller dimensions, excellent elasticity and deformability, and improved skin permeability, which may be due to the synergistic interaction between ethanol and surfactants, thereby promoting the rearrangement of the lipid bilayer of these vesicles [41]. Niu et al. investigated the mechanism of skin penetration of ethosomes and found that ethosomes were able to disturb the lipid structure of the SC, lyse intercellular lipids, and alter the phase transition temperature of the SC. A portion of the ethosomes binds to the SC, while the other portion crosses the intercellular space and cleaves to release the drug during penetration of the SC [42].

Ethosomes have a higher percentage of drug capture compared to rigid conventional liposomes. With the high concentration of ethanol, ethosomes can act as natural preservatives with excellent stability under specific storage conditions [43]. Moreover, ethosomes are soft malleable vesicles that facilitate effective penetration of drugs into the skin, resulting in local and systemic effects [80,81]. Allergic reactions may occur in individuals who are allergic to ethanol or any other component of ethanol, which poses some safety concerns in practical application.

### 3.2. Lipid-Based Nanocarriers

There are two main types of lipid nanoparticles and the main difference lies in their composition, i.e., when composed of solid lipids only, they are called solid lipid nanoparticles. When the composition includes a portion of liquid lipid (usually oil) in addition to solid lipid, they are called nanostructured lipid carriers.

#### 3.2.1. Solid Lipid Nanoparticle (SLN)

SLNs were introduced in 1991 as a better carrier system than conventional colloidal systems with good biocompatibility, storage stability, and high drug encapsulation efficiency [82]. SLNs consist of the main components, i.e., solid lipids, emulsifiers, and water or other solvents. SLNs are a colloidal carrier system consisting of lipid cores with a high melting point and coated with a liquid surfactant. The concentration of the surfactant, which is about 0.5–5%, improves the dispersion stability of SLNs [44]. In addition, the dense lipid matrix decelerates lipid digestion, thus allowing the release of encapsulated compounds in a more sustainable or long-lasting manner [83].

#### 3.2.2. Nanostructured Lipid Carrier (NLC)

Typically, the ingredients in NLC formulations consist of solid lipids, liquid lipids, surfactants, and co-surfactants. NLCs are a further development of SLNs. They are developed or modified by mixing solid and liquid lipids to increase the loading of drugs in the lipid matrix, thereby preventing leakage in the lipid matrix and regulating the release of drugs [45,46]. These advantages are helpful in increasing the hydration of the skin with an occlusive effect, thus improving the penetration capacity of the loaded drug [84].

SLNs and NLCs have received particular attention due to their myriad applications and benefits, exhibiting low toxicity, high biocompatibility, and sustained release behavior [85]. Moreover, they can protect unstable and sensitive drugs from degradation and are able to improve the bioavailability of loaded drugs as well as achieve the targeted delivery of drugs to specific sites [86,87]. However, SLNs and NLCs can present certain difficulties when developing formulations due to their modest loading capacity, especially for more hydrophilic or polar drugs. In addition, there is a risk of drug efflux from the lipid matrix due to changes in the polycrystalline nature with storage time. Meanwhile, a relatively high amount of water is required to produce lipid nanoparticle dispersions, which may increase the risk of microbial contamination. Because of this, preservatives are added during preparation, which might induce side effects in turn [88].

### 3.3. Emulsion-Based Nanocarriers

Microemulsions and nanoemulsions are lipid-based drug delivery systems with the potential to increase drug penetration into the skin [89]. Although they are isotropic dispersions of two immiscible liquids (oil and water), there are significant differences between microemulsions and nanoemulsions.

#### 3.3.1. Microemulsion

In 1943, Hoar and Shulman proposed a definition of microemulsion, describing it as a spontaneously formed transparent or semi-transparent system of water, oil, and a mixture of a substantial amount of surfactants and co-surfactants (generally alcohols of medium chain length) [90]. It is a thermodynamically stable isotropic liquid, microscopically composed of droplets of one or both liquids stabilized by a surfactant interfacial film. Compared to emulsions, microemulsions have smaller droplet sizes, usually within 10–100 nm [91]. Co-surfactants are added to microemulsions to help lower the tension in the surfactant film, creating a more flexible and dynamic flow layer [47].

#### 3.3.2. Nanoemulsion

Nanoemulsions generally refer to a homogeneous system formed by the addition of two immiscible phases (oil and water) through the addition of a suitable emulsifier. The droplet size of nanoemulsions is typically between 20 and 300 nm with a narrow particle size distribution. Stabilization is usually carried out with an appropriate surfactant [92]. Researchers have reported the topical delivery of drugs by nanoemulsions that can enhance skin penetration by altering the lipid structure and permeability gradient of the SC [48].

Both microemulsions and nanoemulsions are carriers, which due to their combined properties, are able to act as penetration enhancers for the skin, thus overcoming the barrier of the SC [49]. Microemulsions and nanoemulsions have similar macroscopic properties, whereas microemulsions are highly thermodynamically stable. Despite having unstable thermodynamics, nanoemulsions are kinetically stable, which makes them resistant to degradation issues. The high thermodynamic as well as kinetic stability enables these systems to increase skin permeability and drug delivery efficiency, thus successfully overcoming the barrier challenges of transdermal delivery, which makes them good vehicles for transdermal drug delivery [50].

### 3.4. Polymeric Nanocarriers

#### 3.4.1. Polymeric Nanoparticle (PNP)

PNP has nanoscale dimensions (1–1000 nm) and the drug is encapsulated within a polymer core or uniformly dispersed in a polymer matrix [93]. PNPs can be divided into two groups according to their morphology and structure: nanocapsules and nanospheres. The former has an oily core where the medication can dissolve, while the latter is surrounded by a polymer shell. Nanospheres are based on a continuous network formed by polymers and the drug is retained inside to be dissolved, encapsulated, or adsorbed on its surface. These two types of PNPs are considered reservoir systems (nanocapsules) and matrix systems (nanospheres) [51]. As drugs are adsorbed onto the surface of PNPs, it usually releases the drugs in a burst first, then gradually and continuously from the polymer matrix or core [93]. A possible mechanism by which PNPs improve skin permeation has been found to be by disorganizing the structure of SC keratin and changing lipid accumulation and lipid structure to a more ordered and dense conformation [52].

PNP has great potential for topical delivery of a wide range of drugs by improving its physicochemical properties, preventing premature degradation, allowing sustained release, maximizing penetration through the SC, and boosting its retention in various skin layers (i.e., the SC, epidermis, and dermis) [94].

#### 3.4.2. Polymeric Micelle (PM)

PM has been extensively studied since the 1990s and even since the late 1980s [95]. PMs are nanoscale drug delivery systems, usually between 10 and 100 nm, formed by the self-assembly of amphiphilic block copolymers with hydrophilic and hydrophobic blocks in aqueous solution. They are typically spherical in shape, characterized by a hydrophobic core with a hydrophilic shell. The self-assembly of PMs is a reversible process that depends on the critical micelle concentration (CMC), which is described as the minimum concentration of the polymer in solution that leads to micelle formation [96]. The micelle concentration is stable above the CMC, but when diluted below the CMC, the micelles decompose [97].

PMs can improve the penetration of hydrophobic drugs into the skin [98]. Possible mechanisms include improving the dissolution of drugs into the skin, increasing the distribution of hydrophilic drugs in the SC, localizing the drug into the hair follicles and keratinocytes in different epidermal layers, and providing storage into the skin by slowly and sustainedly releasing the drug from intact PMs [53]. Despite its many advantages, the large-scale production of PMs remains a challenge based on the scalability and reproducibility of the copolymer and drug-carrying micelles with scalable and reproducible manufacturing processes [99].

#### 3.4.3. Dendrimer

Dendrimers are monomolecular, monodisperse, synthetic spherical polymers with a dendritic branched chain structure. They are appealing for a variety of promising biological applications due to their well-controlled size (3–10 nm), ease of functionalization, high water solubility, clearly defined chemical structure, and biocompatibility [100]. As polymeric skin permeation enhancers that can boost medication transport through the skin, dendrimers have received much attention. Studies have reported that dendrimers facilitate drug penetration by improving the dispersion and diffusion of drug molecules in the skin and making the medication more soluble [54]. However, there are still some issues that need to be addressed in terms of translation to clinical trials. Although dendrimers are attractive for clinical applications due to their distinct structures, the synthesis of these molecules requires cumbersome steps and is therefore both complex and expensive for scaled-up production. In addition, to date, spatial site resistance at the periphery of dendrimers limits the molecules to relatively low generations, thus limiting the extent of amplification that can be achieved [101].

### 3.5. Inorganic Nanoparticles

Inorganic nanostructures are widely applied in drug delivery for cancer therapy with their probable bio-imaging and phototherapy potential. Some nanoparticles with positive charge, high surface lipophilicity, and small size have transdermal capability by passively penetrating SC [56].

Gold nanoparticles (AuNPs) are well explored as a transdermal drug delivery system because of their low cytotoxicity and controllable particle size [57]. AuNPs can be used for both local and systemic transdermal drug delivery. Because of the interaction with skin lipids, GNPs of much smaller sizes (e.g., ~10 nm) can induce transient openings in the organized stratum corneum, accompanied by their penetration into the skin [33]. Furthermore, surface ligands bind with the gold nanoparticles with Au-S bonding, which means various ligands could be conveniently conjugated on the surface of particles by pre-thiolation [58]. Koushki et al. introduced a dendritic cell-specific aptamer for the modification of allergen-loaded AuNPs. The targeted allergen delivery system produced greater epicutaneous immunoregulation in comparison to the non-targeted ones, and co-administration with skin-penetrating peptides led to a more significant effect [102].

Some other metallic particles have also been observed for transdermal application. Fe_3_O_4_ nanoparticles with a pH-sensitive amide bond have successfully penetrated into the deeper dermis via the transfollicular route [103].

### 3.6. Inclusion Complexes

Cyclodextrins (CD) are cyclic oligomers obtained from starch by enzymatic degradation and were discovered in 1891 by the French pharmacist Villiers [59]. It is a cyclic oligosaccharide formed from α-1,4-linked glucose units, where α-CD, β-CD, and γ-CD are the most common natural forms, containing six, seven, and eight glucose units, respectively [60]. Cyclodextrins have the remarkable capability of establishing supramolecular host–guest interactions because of their toroidal shape and non-polar inside [61]. CD’s unique chemical structures allow them to entrap poorly soluble drugs, such as antioxidants, leading to the significant enhancement of drug solubility and stability [62]. Inclusion complexes are formed when the “guest” molecule, usually a drug, is partially or fully included inside the “host’s cavity” [104]. Owing to the hydrophobic cavity, cyclodextrins as ghosts offer the guest a suitable environment for interaction.

Due to increased solubility as well as direct action on the stratum corneum, CD complexes promote a drug’s availability and delivery through the cutaneous barrier [63]. Chulurks S et al. reported that after 30 min, the amounts of nicotine that permeated through pig skin from the nicotine/βCD and nicotine/MβCD inclusion complex gels were nine times as much as pure nicotine gel [105].

## 4. Nanocarrier-Based Transdermal Delivery Technology for Dermatological Therapy

**Figure 3:**
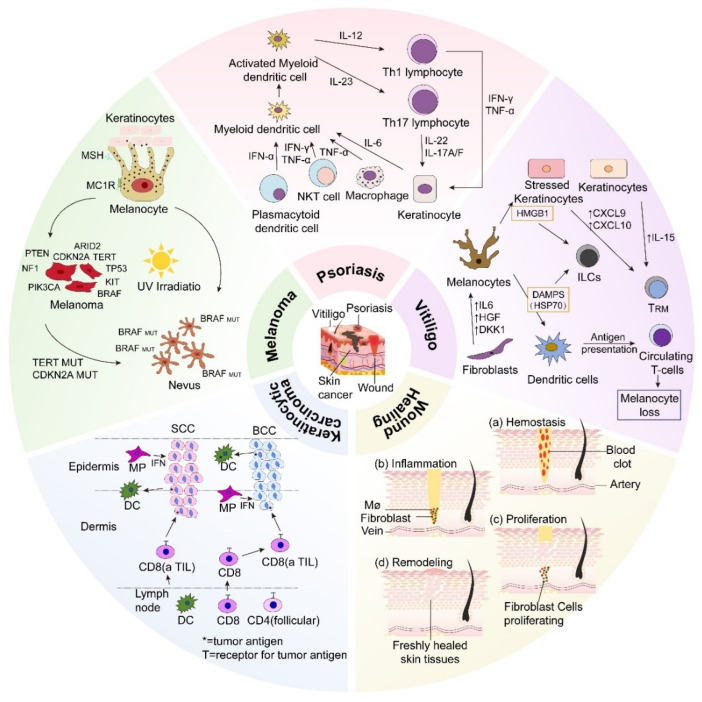
Transdermal drug delivery technologies for dermatoses.

Transdermal delivery technology, as a non-invasive drug delivery system, has received widespread attention in recent years for dermatological therapy. Transdermal delivery technology can deliver drugs to the skin through topical administration. In the treatment of common dermatoses (such as psoriasis, vitiligo, skin cancers, etc.), it has a more ideal effect compared to oral and injectable administration [106]. In addition, transdermal drug delivery can also deliver medications into the bloodstream to treat various diseases [107]. Compared to traditional methods such as oral, intravenous, and subcutaneous injection, it can avoid first-pass metabolism and gastrointestinal side effects, offering the advantages of simplicity, convenience, and high patient compliance [108]. With the advancements in molecular biology and the development of novel delivery systems, transdermal delivery technology has shown great potential and advantages in dermatological treatment (Figure 3). This article reviews transdermal drug delivery technologies used for dermatoses in the past five years (Table 2).

### 4.1. Psoriasis

Psoriasis is prevalent worldwide, affecting the quality of life of a large number of patients [138]. As an immune-mediated chronic inflammatory dermatosis, psoriasis is characterized by hyperkeratosis and parakeratosis of the epidermis, thickening of the spinous layer, vascular dilation, and pinpoint bleeding [139,140]. These symptoms can cause pain, discomfort, and psychological distress, significantly reducing the quality of life for patients.

The pathogenesis of psoriasis involves a complex inflammatory cascade, with various innate immune cells (keratinocytes, dendritic cells, NKT cells, and macrophages) and signal transduction pathways participating in the disease’s development [141]. The onset and progression of psoriasis are influenced by a combination of environmental, genetic, and immune factors, which complicates its diagnosis and treatment [142]. Common clinical treatments include traditional systemic drug therapy, topical drug therapy, phototherapy, and biological agents [143]. Currently, drugs commonly used for the treatment of psoriasis include MTX and some biologics [144]. Although MTX has a certain efficacy in treating psoriasis, its long onset time, liver toxicity, and tendency for relapse after discontinuation limit its widespread use. Additionally, since psoriasis is typically a lifelong disease requiring repeated interventions, transdermal delivery technology can provide a treatment method that is highly safe and effective and has low side effects.

**Figure 4:**
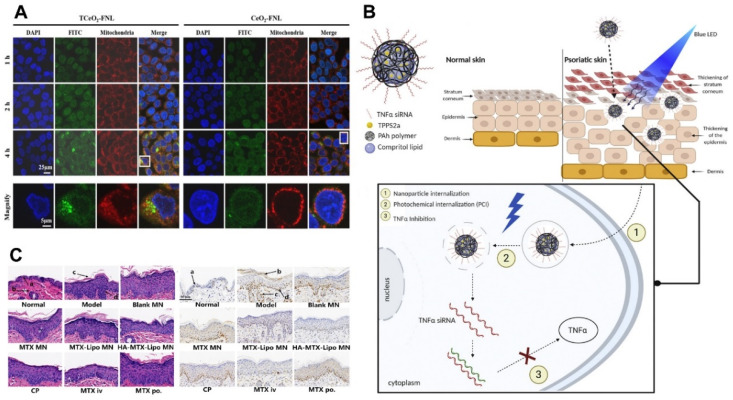
Schematic illustration and characterization of nanoparticles for psoriasis treatment. (**A**) CLSM images of HaCaT cells treated with TCeO_2_-FNL/CeO_2_-FNL at predetermined time points (red: mitochondria; green: TCeO_2_-FNL/CeO_2_-FNL). Reprinted from Ref. [110]. (**B**) Scheme of the nanoparticle PLN-TPPS2a-TNF siRNA and PCI mechanism. Reprinted with permission from Ref. [111]. Copyright 2021 Elsevier. (**C**) H&E sections and immunohistochemical micrographs of skin of psoriasis mice model treated with HA-MTX-Lipo MN (Scare bar: 50 μm). Reprinted with permission from Ref. [112]. Copyright 2024 Elsevier.

Transdermal delivery technology based on nanomedicines can overcome the drawbacks of traditional topical drug delivery strategies, such as poor transdermal efficiency and ineffective treatment [145]. Yu et al. developed a peptide-modified Cur-loaded liposome (CRC-TD-Lip) to accelerate the transdermal delivery of Cur and improve the inhibition of psoriasis. The mRNA expression levels of IL-17A, IL-17F, IL-22, and IL-1β in the CRC-TD-Lip of mice were decreased 4- to 6-fold compared with those in psoriatic mice, which experimentally confirmed the enhanced transdermal ability of CRC-TD-Lip [109]. Wang et al. prepared a CeO_2_ flexible nanoliposome modified with TRA and TPP, called TCeO_2_-TRA-FNL [110]. In vitro EGF-induced and H_2_O_2_-induced models showed that TCeO_2_-TRA-FNL effectively reduced the inflammation level of HaCaT cells and attenuated oxidative stress. TCeO2-TRA-FNL-Gel treatment resulted in a 3-fold reduction in erythema, scaling, and skin thickness scores in mice compared to the Free-TRA group, showing promising therapeutic effects on psoriasis (Figure 4A). Suzuki et al. developed an siRNA delivery system based on hybrid polymer-lipid nanoparticles (PLN-TPPS2a-TNF siRNA) and combined it with photochemical internalization as a topical formulation for psoriasis treatment (Figure 4B) [111]. In vitro delivery studies using a porcine skin model showed that PLNs successfully delivered siRNA and photosensitizers into the skin. In vivo experiments confirmed that a 1.38-fold knockdown of TNFα was observed with PLN-TPPS2a-TNFα siRNA compared to the psoriasis group, suggesting that it enhanced the preventive effect of psoriatic plaques. Solid lipid nanoparticles loaded with cyclosporin A deliver approximately 1.0 mg of cyclosporin A to the skin with reduced transdermal permeation, indicating its promising topical administration [115].

Transdermal delivery systems also have potential applications in the systemic treatment of psoriasis. To enable effective drug penetration through the hyperkeratotic skin of psoriasis patients and enhance therapeutic efficacy, Shen et al. developed HA-modified liposomes loaded with MTX and incorporated them into microneedles (HA-MTX-Lipo MNs) [112]. The results showed that HA-MTX-Lipo MNs inhibit the progression of psoriasis and reduce erythema, scaling, and thickening of the skin by down-regulating the expression of mRNA levels of pro-inflammatory cytokines IL-23 and TNF-α (Figure 4C). Shah P et al. used niosomes to deliver Desoximeta-sone, which can be used to treat a variety of skin conditions such as allergic reactions, eczema, and psoriasis. Desoximeta-sone loaded into niosomes increased the skin permeability of Desoximeta-sone compared to the raw drug [113]. He E et al. developed a microemulsion-based drug delivery system for transdermal delivery in order to improve the efficacy and permeability of indirubin. The In Vitro Skin Permeation and Deposition Study showed that the accumulated drug exudation in the final formulation was 2.1-fold and 13.1-fold higher than that of the oil solution and the aqueous solution, respectively; both the permeation and retention of the skin increased. This preparation can improve psoriasis symptoms by down-regulating the expression of IL-17A, Ki67, and CD4+T, providing great scalability for researchers to increase the concentration of targeted drugs [114]. Chamcheu J et al. developed chitosan-based nanoformulated (-)-epigallocatechin-3-gallate (EGCG). In the imiquimod-induced psoriasis-like dermatitis model in mice, nanoEGCG showed a 20-fold dose advantage over free EGCG, representing a promising drug delivery strategy [116].

### 4.2. Vitiligo

Vitiligo is a common disorder with a global incidence of 0.5–2.0% [146]. Vitiligo is a condition in which the skin develops white patches due to melanin deficiency as a result of the destruction of melanocytes in the skin. The most common form is non-segmental vitiligo, which is clinically characterized by well-defined, milky-white, depigmented patches. The occurrence of vitiligo affects males and females equally but is more pronounced in people of color [147]. Vitiligo is not contagious or life-threatening but may seriously affect the physical and mental health of the patient.

The pathogen of vitiligo remains unclear, and it is commonly believed that it occurs under the combined influence of genetic, autoimmune, and environmental factors [148]. The etiology of vitiligo mainly involves the loss or destruction of functional melanocytes in the hair follicles and skin [149]. Autoantigens are released from melanocytes in vitiligo patients due to mitochondrial failure and increased cellular stress, which triggers both innate and adaptive immunity [150]. Vitiligo autoimmunity is influenced by the expression of the Fc-γ receptor on melanocytes and a protein that inhibits macrophage migration [151]. In addition to causing abnormal immune responses against these dysfunctional melanocytes, the combination of genetic factors and cellular flaws also results in the self-recognition of melanocyte antigens. In addition, the hyperimmune reaction to melanocytes is facilitated by malfunctioning regulatory T cells, leading to the onset and progression of vitiligo [152].

**Figure 5:**
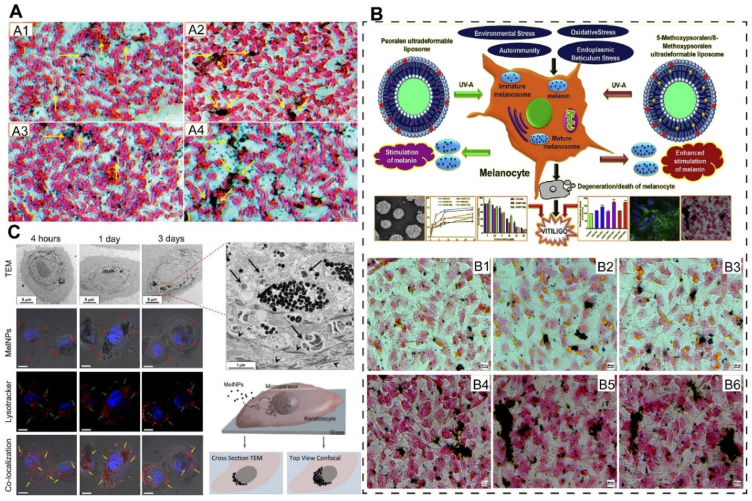
Schematic illustration and characterization of nanoparticles for vitiligo therapy. (**A**) Using Fontana–Masson silver staining, the B16F10 cell line’s melanin pigment is visible. Dark black staining of the melanin in various groups is shown by yellow arrows: (**A1**) Control, (**A2**) PSR-UDL, (**A3**) RSV-UDL, and (**A4**) PSR + RSV-UDL (Scare bar: 1000 μm). Reprinted with permission from Ref. [117]. Copyright 2017 Elsevier. (**B**) Schematic illustration of 5-MOP/8-MOP sol and Fontana–Masson silver staining to visualize melanin pigment in B16F10 cells where melanin is stained dark black in different groups: (**B1**) PSR Sol, (**B2**) 5-MOP Sol, (**B3**) 8-MOP Sol, (**B4**) PSR UDL, (**B5**) 5-MOP UDL, and (**B6**) 8-MOP UDL. Reprinted with permission from Ref. [119]. Copyright 2019 Elsevier. (**C**) The uptake, transport, and accumulation of MelNPs in HEka cells were analyzed using transmission electron microscopy and confocal optical microscopy. Reprinted from Ref. [120].

The currently available therapies for vitiligo include phototherapy and immunosuppression. However, treatment outcomes are unsatisfactory with a wide range of side effects, and therefore no drugs have received FDA approval to be used in vitiligo [153]. Several studies have been conducted on the transdermal delivery of drugs using nanocarriers for vitiligo. Doppalapud S et al. developed super deformable liposomes loaded with PSR and resveratrol, which could act on vitiligo via a dual mechanism of stimulating tyrosinase activity and scavenging free radicals (Figure 5A). Approximately 1.75-fold and 2.45-fold increases in melanin content were observed with the first and second applications of the final formulation compared to the control group, demonstrating a significant stimulation of melanin and tyrosinase activity by liposomes [117]. Saxena et al. prepared liposomes loaded with NSO and VSO using the thin film dispersion method and loaded them in gels [118]. The drug release after 24 h was found to be 76.18% (NSO) and 73.12% (VCO) for liposomal dispersions, achieving a slow drug release. In vitro and in vivo experiments showed that the prepared liposomal gels significantly prolonged the penetration of the drug into the skin and its accumulation at the disease site and improved its effectiveness in the treatment of vitiligo. Mahira S et al. prepared liposomes encapsulated with PSR and its derivatives 5-MOP and 8-MOP in Edge-activated ultradeformable liposomes (UDLs). 5-MOP UDL and 8-MOP UDL showed enhanced stimulation of melanin levels by increasing melanin 1.97-fold and 1.86-fold after 72 h of incubation compared to the control group (Figure 5B) [119]. Huang et al. prepared melanin-like nanoparticles (MelNPs) through the spontaneous oxidation of dopamine and studied their absorption, transport, distribution, and ultraviolet (UV) protection capabilities in human keratinocytes [120]. Experiments showed that in human epidermal keratinocytes, MelNPs were endocytosed, underwent perinuclear aggregation, and formed a supranuclear cap, mimicking the cellular distribution and function of real melanosomes. This suggests their potential application in vitiligo treatment (Figure 5C).

### 4.3. Wound Healing

The integrity of the skin is crucial for human safety since it serves as the body’s first line of defense against external microorganisms and viruses [154,155]. Patients suffer discomfort, including pain, bleeding, inflammation, delayed healing, scar formation, and even death, when the integrity of their skin is damaged [156]. Wound healing is a fundamental stage in the reconstruction of skin damage and is usually divided into four successive and potentially overlapping processes: hemostasis, inflammation, proliferation, and remodeling [157,158].

The first is the hemostatic phase, in which small blood vessels and capillaries surrounding the wound reactively constrict to reduce local blood flow [159]. Subsequently, platelets are attracted to the exposed collagen fibers to aggregate into a blood clot. Simultaneously, platelets release prostaglandins and 5-hydroxytryptamine, two vasoactive chemicals that further narrow blood vessels and decrease blood flow. Finally, endogenous and exogenous coagulation processes are initiated [160]. The inflammatory phase that follows is marked by enhanced vascular permeability and activated inflammatory cells, including neutrophils, lymphocytes, and monocytes, moving into the wound in response to chemokines. The proliferative phase is closely related to the inflammatory phase and is an important step in re-establishing the skin barrier function [161]. Keratinocytes, fibroblasts, and vascular endothelial cells are the key cell types that are involved in the skin repair process. Through their proliferative and migratory activity, these cells achieve neovascularization, granulation tissue development, and wound epithelial regeneration. The remodeling phase involves the maturation and reconstruction of neoplastic tissue and is the final stage of wound healing [162]. This phase, which can extend for months or years, primarily entails the reorganization of collagen, the regression of enlarged capillaries, and the breakdown of superfluous collagen fibers by collagenases. Eventually, normal connective tissue develops from the granulation tissue created during wound healing [163]. Studies have confirmed that temperature, pH, and other factors affect these processes [164].

By absorbing osmotic fluids and isolating wounds from the external environment, conventional wound dressings (such as gauze, cotton wool, and bandages) stop bleeding and shield wounds from bacterial infection. However, they lack antibacterial or anti-inflammatory properties and can even impede the healing process [165]. Ideal wound dressings should have the following characteristics: good biocompatibility, antimicrobial activity, water absorption, water retention, no cytotoxicity, and good biodegradability [166,167,168].

**Figure 6:**
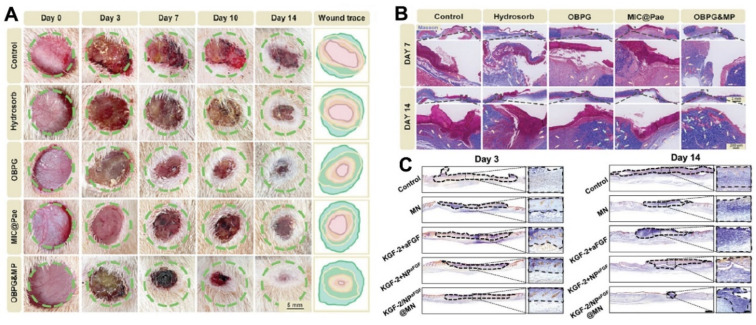
(**A**) Representative images of the wound healing trace after the treatment of OBPG&MP NPs (Scar bar: 5 mm). Reprinted with permission from Ref. [121]. Copyright 2024 Wiley. (**B**) Histological evaluation of the regenerated skin via Masson staining treated with OBPG%MP NPs (Scar bar: 2 and 200 µm). Reprinted with permission from Ref. [121]. Copyright 2024 Wiley. (**C**) Representative images of immunohistochemistry staining of cytokeratin of wound tissues after the treatment of KGF-2/ aFGF-NPs MNs on Day 3 and Day 14. Reprinted with permission from Ref. [124]. Copyright 2024 Wiley.

The nano transdermal delivery system has excellent efficacy in the anti-inflammation of wounds. To keep the area moist and decrease the risk of infection while hastening the healing process, a study by Zhang et al. introduced a stimuli-responsive glycopeptide hydrogel (OBPG&MP) constructed from hyacinthine polysaccharides, gallic acid-grafted ε-polylysine, and micelles loaded with paeoniflorin [121]. With each release of therapeutic chemicals, the hydrogel responded to the inflammatory microenvironment of chronic wounds by eliminating bacterial infection, neutralizing ROS, modifying macrophage polarization, suppressing inflammation, and promoting vascular regeneration and extracellular matrix remodeling. Studies conducted in vivo and in vitro have shown how effective OBPG&MP is at modifying the wound microenvironment and promoting skin tissue regeneration and remodeling in chronic wounds (Figure 6A,B). Yang et al. prepared propolis nanoparticles (PNPs) using the pH difference method and characterized them. In the full-thickness skin defect model of mice, compared with the wounds of other groups, the wound healing of PNP treatment was 3–4 days faster. Histological observation showed that the wounds treated with PNPs had tissue epithelium, hair follicles, and dense collagen fibers, indicating that PNPs have the potential to become an ideal choice for wound-healing applications [122]. Kazemi M et al. prepared a piroxicam plasmid and combined it with iontophoresis therapy, which significantly enhanced the permeability of the piroxicam plasmid and had a significant inhibitory effect on the inflammatory response of wounds [123]. The dihydromyricetin-loaded inclusion complex significantly reduced the M1 phenotypic transition in RAW264.7 cells, effectively restoring M2 polarization, thereby shortening the inflammatory period. The final formulation exhibited superior free radical scavenging performance (82.95 ± 0.94% and 93.88 ± 0.82% for ABTS and DPPH radical scavenging activities, respectively), making them excellent candidates for promoting wound healing [128].

Skin infection is a key concern in the management of wound healing. He et al. fabricated a novel microneedle patch for the continuous transdermal delivery of keratinocyte growth factor-2 (KGF-2) and nanoparticles loaded with aFGF, and the results showed that the microneedle could successfully penetrate through the charred crusts of the burn wounds, release KGF-2 and aFGF-NPs sequentially, enhance the collagen deposition, increase neovascularization, and have a preventive effect on the deterioration of burns (Figure 6C) [124]. To treat bacterially infected cutaneous wounds, Qin et al. produced a novel functional MN array made of methacrylated hyaluronic acid embedded with pH-responsive functionalized zeolitic imidazolate framework-8 nanoparticles [125]. In vitro antimicrobial experiments demonstrated that 3.0 mg/mL of nanoparticles reduced the number of colonies of *S. aureus* and *P. aeruginosa* from approximately 9.5 to 8.5 log_10_CFU/mL compared to the control. In addition, it can have a significant positive therapeutic effect on the healing of bacterial infected skin wounds by up-regulating HIF-1α expression and promoting wound angiogenesis in a synergistic manner.

In addition, transdermal delivery systems can enhance wound healing by improving drug penetration and retention. The DFO-loaded transferosomes were found to be effective in sustaining DFO release, which complied with zero-level dynamics [126]. Gupta P et al. solved the problem of the poor water solubility and low bioavailability of hesperidin by incorporating it into the kernel of a dendritic polymer. Rat skin treated with hesperidin showed the maximum intensity at the skin surface of 0–5 μm, while dendrimer showed the deposition of the drug in the epidermis up to 15–25 μm. By improving the skin permeability of the drug, the dendritic polymers showed the best overall performance in improving all-over wounds [127].

### 4.4. Skin Cancer

Skin cancers, which typically include melanoma, basal cell carcinoma, and squamous cell carcinoma, are often treated with surgery, phototherapy, immunotherapy, and chemotherapy. For many years, surgical excision has been the recommended course of treatment for patients with skin cancers. Even though excision is usually quite effective, a number of factors, such as patient problems, the lesion’s anatomical position, and the patient’s possible sensitivity to multiple cycles of therapy, may affect whether or not surgery can proceed [169]. Therefore, topical and transdermal treatments may be more appropriate options, allowing for better therapeutic levels at the site of action and reduced toxicity.

The most commonly used topical agents for the treatment of skin cancer are 5-fluorouracil, imiquimod, sonidegib, and dacarbazine. However, they are usually associated with serious side effects such as allergic reactions, hypersensitivity reactions, and severe pain. Moreover, the physicochemical properties of the drugs and the physiological barriers of the skin limit the anticancer efficacy of topical and transdermal delivery. The application of transdermal delivery technologies in medicine offers a unique opportunity to improve current therapeutic approaches to cancer and other diseases.

#### 4.4.1. Melanoma

Melanoma is one of the fastest-growing malignant tumors, caused by the rapid proliferation of melanocytes (pigment-producing cells of the skin), and is characterized by a low survival rate, high metastasis, quick recurrence, and malignant proliferation [170]. Melanomas on sun-damaged skin are further divided into low and high cumulative sun damage (CSD) depending on the surrounding skin’s histology of CSD. Low-CSD melanomas include superficial diffuse melanomas and high-CSD melanomas include malignant nevus of freckle-like nevus and connective tissue-promoting proliferative melanomas. Non-CSD-associated melanomas include melanoma of the extremities, mucosal melanoma, congenital or blue nevus melanoma, and uveal melanoma [171].

**Figure 7:**
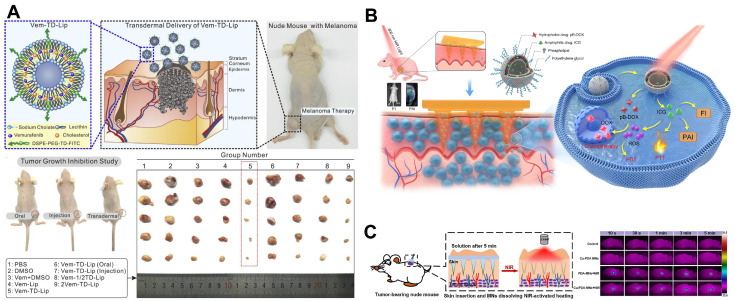
(**A**) Preparation and in vivo antitumor evaluation of Vem-TD-Lip. Reprinted with permission from Ref. [130]. Copyright 2018 Elsevier. (**B**) Design principle of the NIR light-activatable dissolving MN system (MN-pB/I) for multimodal theragnostic application in melanoma. Reprinted with permission from Ref. [131]. Copyright 2023 Springer Nature. (**C**) Scheme and in vivo antitumor evaluation of Cu-PDA-based synergistic comprehensive treatment for melanoma tumor model in Balb/c mice. Reprinted with permission from Ref. [132]. Copyright 2021 Elsevier.

The application of nanocarriers is a promising therapeutic strategy. ICG has been used clinically, but the use of ICG is hampered by the instability of aqueous solutions. Lee et al. prepared ICG liposomes coated with chitosan, which showed a 2-fold total ICG recovery (compared to free ICG) after a 12 h skin penetration study. The final formulation significantly increased the cellular uptake and photocytotoxicity of ICG in B16/F10 melanoma cells and also greatly improved the skin permeability of ICG, which has a potential application in topical melanoma PDT [129]. By preparing peptide TD-modified vemurafenib-loaded liposomes (Vem-TD-Lip), Zou et al. demonstrated that in male mice, transdermal administration of Vem-TD-Lip effectively targeted and inhibited subcutaneous melanoma and that in terms of minimizing major organ damage, dermal administration of Vem-TD-Lip was superior to oral and intraventricular administration (Figure 7A) [130]. This work provided a new strategy for targeting and inhibiting subcutaneous melanoma with vemurafenib.

Repetitive, multimodal therapy is often necessary to treat melanoma because of the disease’s aggressive and recurrent nature. Liu et al. designed a near-infrared photoactivatable soluble polyvinylpyrrolidone MN array (MN-pB/I) (Figure 7B), which contained ICG- and ROS-activatable doxorubicin precursor (pB-DOX) co-loaded liposomes. The growth of the tumors in the liposomes was inhibited by 93.5%, providing a promising candidate for clinical melanoma therapy [131]. Song et al. prepared copper-doped polydopamine nanoparticles (Cu-PDA NPs) and loaded them into MNs for the synergistic treatment of cutaneous melanoma via photo-thermal and chemo-kinetic treatment. Polymeric nanoparticle accumulates the high photothermal effect of Cu-PDA NPs (~50.40%) to acquire the energy from NIR irradiation. A B16/F10 mouse Melanoma model showed that the active delivery of Cu-PDA NPs greatly inhibited tumor proliferation and induced mixed necrosis/apoptosis of tumor cells in vivo (Figure 7C) [132].

#### 4.4.2. Keratinocyte Carcinoma

The primary cells found in the skin’s epidermal layer, keratinocytes, are the source of keratinocyte carcinoma (KC), a kind of skin cancer. KC consists of two subtypes, basal cell carcinoma (BCC) and squamous cell carcinoma (SCC). Of these, BCC is the most common skin cancer, accounting for 80% of keratinocyte-derived carcinomas [172].

BCC is primarily seen on the dorsum of the hands and face in middle-aged or older patients and is believed to originate from follicular stem cells in the epidermis. Environmental, phenotypic, and genetic factors interact in a complicated way to determine the risk of developing basal cell carcinoma [173]. It is believed that UV radiation is a major factor in the formation of BCC.

SCC is generally considered to develop from precancerous lesions called actinic keratoses (AKs) [174]. The greatest risk factor for the development of AK and cSCC is UV exposure. Therefore, the head and neck region, which is heavily exposed to UV, is the most common site of development. A report by the World Health Organization (WHO) showed that there are an estimated 2–3 million cases of KC worldwide each year [175]. Therefore, the development of effective treatments is necessary. Kandekar et al. developed polymeric micellar gels loaded with the hedgehog pathway inhibitor VSD, which were shown to significantly increase the aqueous solubility and stability of VSD and were able to efficiently penetrate the epidermis of the skin, which allows for targeted localized topical BCC topical therapy [133]. Darade et al. developed a micelle-based formulation of TAK-441 using D-α-tocopherol polyethylene glycol 1000 succinate, which resulted in a notable increase in the skin deposition of TAK-441 after 12 h. TAK-441 is also a powerful inhibitor of the hedgehog pathway [134]. Skin biodistribution profiles indicated that TAK-441 was delivered predominantly to the surviving epidermis and upper dermis, with almost negligible transdermal penetration, thus reducing the risk of systemic side effects in vivo. 5-Aminolevulinic acid has been used for the treatment of skin cancer and other tumors due to its safety and cost effectiveness; however, poor permeability and targeting limit its application. Bacillus licheniformis was used by Shivashankarappa et al. to produce silver nanoparticles. The nanoparticles dramatically slowed the growth of B16/F10 and A431 (human skin squamous carcinoma cells) in vitro [176].

### 4.5. Others

In addition to the above-mentioned techniques, transdermal drug delivery techniques have a wide range of applications in atopic dermatitis (AD), eczema, hyperplastic scarring, etc. AD is a chronic, recurrent dermatological disorder that shortens life expectancy dramatically. Multiple processes, such as immunological dysregulation, skin inflammation, and alteration of the skin barrier, may play a role in the pathogenesis of AD. Zhu et al. developed novel nanoparticles embedded with Cur and coated with filaggrin. The final formulation markedly elevated the skin permeability and cumulative turnover of transferred Cur, which provided a greater than a 3.8-fold increase relative to free Cur. It was shown to deliver Cur topically to penetrate the dermis and exert an antidermatitic effect by inhibiting the nuclear translocation of NF-κBp65, thereby down-regulating the production of inflammatory cytokines and chemokines in keratin-forming cells [135]. Abuelella et al. fabricated nanoparticles containing chitosan and HA polyelectrolyte composite nanoparticles for the delivery of ETX, which were able to significantly improve the skin penetration of the drug and exhibited excellent anti-inflammatory properties in dithranol-induced ear dermatitis in mice, resulting in good therapeutic efficacy in irritant contact dermatitis and eczema [136]. In addition, Jiang et al. reported a polyhyaluronic acid (PHA)-based spherical nucleic acid for the co-delivery of a typical chemotherapeutic drug DOX and a targeted tissue inhibitor of metalloproteinases 1 (TIMP-1) for the treatment of HS caused by abnormal fibroblast proliferation [137]. It was shown that PHA-based SNAs greatly increased DOX’s cytotoxicity, accelerated cellular absorption, and down-regulated TIMP-1 expression. The value of drug accumulation in the dermis of PHAAR nanoparticles was about 1.7 times higher than that of the free drug moisturizer group, which further proved that nanoparticles could enhance skin penetration and facilitate drug delivery to deeper tissue. Furthermore, in a xenograft mouse model, these nanoparticles reduced the load and progression of hypertrophic scarring and encouraged the apoptosis of hypertrophic scar cells without causing any negative side effects, suggesting that they may be used to treat a variety of skin conditions.

Altogether, transdermal delivery technology shows great potential and value in dermatological treatment and can provide a safe and effective modality to improve the quality of life of patients with dermatoses.

## 5. Conclusions and Outlook

In this review, we delved into the recent advances in transdermal drug delivery technology, providing a comprehensive overview of its applications in the treatment of dermatoses. By carefully selecting appropriate carriers, this technology has significantly enhanced the skin’s ability to absorb and utilize drugs, thereby improving their therapeutic efficacy. We analyzed the advantages and challenges of different transdermal delivery systems, such as vesicular carriers, lipid carriers, and polymeric nanoparticles, in practical applications, and explored the mechanisms of action of various transdermal technologies. In the future, as biotechnology and material science continue to advance, new transdermal delivery systems are expected to revolutionize the way drugs are administered and improve their effectiveness in treating various medical conditions. Meanwhile, the rise of personalized medicine also provides a new opportunity for the development of transdermal drug delivery technology, which is expected to achieve more accurate and customized treatment plans by combining big data and artificial intelligence. In conclusion, while transdermal delivery technology holds immense promise, continued research and optimization play crucial roles in overcoming the existing technical challenges. Advancements in formulation techniques, drug penetration enhancers, and safety evaluations will pave the way for more efficient and safer drug delivery, ultimately benefiting patients with dermatoses.

## Figures and Tables

**Table 1:** Nanocarriers for transdermal delivery.

Category	Composition	Preparation Methods	Advantages	Limitations	References
Liposome	Phospholipids, cholesterol	Thin film hydration method, reverse phase evaporation and solvent injection method, detergent depletion method	Low toxicity, good biocompatibility and biodegradability	Poor stability	[34]
Niosome	Nonionic surfactants, cholesterol	Ether injection method, reverse phase evaporation method, thin film hydration method, microfluidization method, hand-shaking method, the “bubble” method, sonication method, heating method, freeze and thaw method, dehydration rehydration method, proniosome technology	High stability, low toxicity	High temperatures tend to destroy structures	[35,36,37]
Transferosome	Phospholipids, edge activators	Rotary film evaporation, thin film hydration, reverse-phase evaporation, vortexing sonication, ethanol injection, freeze–thaw	Highly elastic, deformable	Skin irritation, low stability	[38,39,40]
Ethosome	Phospholipids, high concentrations of ethanol	Cold injection method, hot injection method, vortex/sonication method, rotary film evaporation	Smaller dimensions, excellent elasticity and deformability	Skin irritation	[41,42,43]
Solid lipid nanoparticle	Solid lipids, surfactants, co-surfactants	High shear homogenization, solvent emulsification/evaporation, spray drying method, micro-emulsion, solvent diffusion and solvent injection, ultrasonication	High stability, low toxicity, good flexibility	Low drug loading; low stability	[44]
Nanostructured lipid carrier	Solid lipid, liquid lipid, surfactants, co-surfactants	The high shear homogenization method, solvent dispersion method, film-ultrasonic method, ultrasonic emulsion evaporation method	High drug-loading capacity, high stability, high biodegradability	Tend to gel	[45,46]
Microemulsion	Water, oil, surfactants, and co-surfactants (generally alcohols of medium chain length)	Polymerization in emulsion method, interfacial polymerization, precipitation of pre-formed polymer, solvent extraction method	Thermodynamic stability	Toxicity	[47]
Nanoemulsion	Oil, water, emulsifiers	Ultrasonication, microfluidic homogenization, rotor-stator homogenization, phase inversion composition, phase inversion temperature, solvent evaporation	Improve solubility, enhanced permeability	Low stability	[48,49,50]
Polymeric nanoparticle	Natural/synthesis polymers	Nanoprecipitation, emulsification-solvent evaporation, emulsification solvent diffusion, salting-out technique, emulsion polymerization, surfactant-free emulsion polymerization, mini-emulsion polymerization, micro-emulsion polymerization	High stability	Difficulties in large-scale production	[51,52]
Polymeric micelle	Polymers preferably, polyethylene glycol, aqueous solution, ligands	Dialysis method, solvent evaporation method, high-pressure emulsification solvent evaporation	Accurate release	Difficulties in large-scale production	[53]
Dendrimer	Core molecule, surface units, monomers	Divergent synthesis, convergent synthesis, combined divergent/convergent synthesis	Increase the solubility of high lipophilic drugs	Difficulties in large-scale production	[54,55]
Inorganic nanoparticles	Inorganic compound	Chemical method, biological method, green synthesis	Low cytotoxicity, controllable particle size	Low biocompatibility	[33,56,57,58]
Inclusion complexes	Cyclodextrins, drugs	Kneading or slurry method, solution or co-precipitation method, solvent evaporation, dry mixture, damp mixing, extrusion	Significant enhancement of drug solubility and stability	Kidney toxicity	[59,60,61,62,63]

**Table 2:** Transdermal drug delivery technologies for dermatoses in the past five years.

Disease	Drug Delivery System	Loaded Drug	Characterization Parameter	Advantages of Nanocarriers			References
				Drug Properties	Advantages of Penetration and Accumulation	Efficacy	
Psoriasis	Liposome	Curcumin (Cur)	94 nm, EE% = 97%	Cur can be incorporated into liposomes, which markedly improves the solubility and stability of the drug	In vitro permeation experiments demonstrated that the skin permeation of curcumin in the liposome group increased by approximately 60% within 24 h and that the enhanced transdermal effect increased with time	In vivo study showed that liposomes had better anti-psoriasis efficacy in terms of skin inflammation scores, visible skin symptoms, skin pathology sections and skin cytokine mRNA levels	[109]
	Liposome	All-trans retinoic acid (TRA), triphenylphosphine (TPP)-modified cerium oxide (CeO_2_)	60–70 nm, EE% > 96%	TRA loaded into liposomal can improve the solubility and irritation of the drug	Liposomal gel further showed sustained drug release behaviors, great transdermal permeation ability, and greater skin retention than the free TRA	Liposomal gel eradicates excess ROS and achieves satisfactory anti-inflammatory and antioxidant capabilities.	[110]
	Lipid nanoparticle	siRNA	142.1 nm, EE% = 99.5%	Loading siRNA in Nanostructured lipid carrier protects siRNA from biodegradation	The results from the skin permeation and retention studies showed that the nanostructured lipid carrier enhanced the retention of siRNA in the skin layers	A 1.38-fold knockdown of TNFα was observed with nanostructured lipid carrier compared to the psoriasis group, suggesting that it enhanced the preventive effect of psoriatic plaques	[111]
	Liposome	Methotrexate (MTX)	200 nm	MTX loaded into liposomal can improve the solubility and irritation of the drug	Skin permeation study indicated the increase in permeation of MTX with liposomal carriers	Liposome-loaded microneedles inhibit the progression of psoriasis and reduce erythema, scaling, and thickening of the skin by down-regulating the expression of mRNA levels of pro-inflammatory cytokines IL-23 and TNF-α	[112]
	Niosome	Desoximeta-sone	374.80 ± 9.48 nm, PDI 0.289 ± 0.01, zeta potential −63.83 ± 4.26 mV	Encapsulation of corticosteroids in an appropriate carrier system can improve therapeutic efficacy and drug targeting by reducing adverse effects and increasing patient compliance	Desoximeta-sone loaded into niosomes increased the skin permeability of Desoximeta-sone compared to the raw drug	Niosome can used to treat a variety of skin conditions such as allergic reactions, eczema, and psoriasis	[113]
	Microemulsion	Indirubin	84.37 nm, PDI < 0.2, zeta potential 0~−20 mV	Indirubin is loaded into microemulsions to increase its solubility and bioavailability	The transdermal flux and skin retention of indirubin at 24 h were 47.34 ± 3.59 μg/cm^2^ and 8.77 ± 1.26 μg/cm^2^, respectively	Results showed that this preparation can improve psoriasis symptoms by down-regulating the expression of IL-17A, Ki67, and CD4+T	[114]
	Solid lipid nanoparticle	Cyclosporine A	216 ± 5 nm	Cyclosporine A is loaded into solid lipid nanoparticle to increase its solubility	Skin permeation studies using pig ear as a model revealed about 1.0 mg of cyclosporine A was delivered to the skin with transdermal permeation.	It can be used for topical administration of cyclosporine A to avoid its systemic side effects	[115]
	Metal nanoparticle	Epigallocatechin gallate (EGCG)	211.3 nm, PDI 0.132	EGCG is loaded into metal nanoparticle to increase its solubility and bioavailability	Results showed that a controlled release rate of EGCG from metal nanoparticle peaked at about 50% within 6 h, approaching a maximal release of 100% after 24 h	Like free EGCG, metal nanoparticle treatment induced differentiation, and decreased proliferation and inflammatory responses in cultured keratinocytes, but with a 4-fold dose advantage	[116]
Vitiligo	Liposome	Psoralen, resveratrol	120–130 nm, EE% > 74%	Enhancement of transdermal permeability of psoralen and solubility of resveratrol by loading psoralen and resveratrol into liposomes	Co-loaded liposome showed 65.11 ± 7.57% release of PSR and 72.56 ± 12.85% release of resveratrol in 18 h	Combination of PSR and resveratrol acts through dual mechanisms of action viz., stimulation of pigmentation and restoration of redox balance by free radical scavenging activity for effective treatment of vitiligo	[117]
	Liposome	Nigella sativa seed oil (NSO), virgin coconut oil (VCO)	206 nm, zeta potential −33 mV	Liposomes improve skin penetration of drugs	The drug release after 24 h was found to be 76.18% (NSO) and 73.12% (VCO) for liposomal dispersions, achieving a slow drug release	The co-entrapment of NSO and VCO into liposomal carriers may offer therapeutic advantages such as controlled release, enhanced drug penetration, and improved efficiency, suggesting its potential as an effective drug delivery system for dermatological problems	[118]
	Liposome	Psoralen (PSR), 5-methoxypsoralen (5-MOP), 8-methoxypsoralen (8-MOP)	PSR UDL: 98.6 nm, 5-MOP UDL: 125.4 nm, 8-MOP UDL: 113.1 nm	PSR loaded into liposomal can improve the irritation of the drug	PSR liposomes showed 73.30 ± 0.41% drug release in 8 h. Results showed that developed UDL carrier has the capability to sustain the release of drugs over prolonged period of time	Data showed that liposomes up-regulated the melanin and tyrosinase levels than other groups at low dose	[119]
	Polymeric nanoparticle	Dopamine	200 nm	Liposomes enhance the efficacy of drugs	No relevant research data in the article	Polymeric nanoparticles with UV photoprotective properties prevent DNA damage and have the potential to be used as artificial melanosomes to develop new therapies	[120]
Wound Healing	Micelle	Paeoniflorin	96 nm, zeta potential −8.4 mV	Paeoniflorin loaded into micelle can improve the solubility and stability of the drug	Data showed a sustained release of paeoniflorin at least 72 h could be achieved	Micelle adapts responsively to the inflammatory microenvironment of chronic wounds, sequentially releasing therapeutic agents to eradicate bacterial infection and suppress inflammation and ECM remodeling, playing a critical role across the inflammatory and remodeling phases of wound healing.	[121]
	Polymeric nanoparticle	Propolis	117 nm, zeta potential −9 mV	Propolis loaded into micelle can improve the stability of the drug	The results showed that propolis has a certain slow-release effect after preparation of nanoparticles	Polymeric nanoparticle affected the expression of several inflammatory mediator genes and antioxidant genes, and significantly accelerated the wound healing process by inducing more blood vessel and collagen formation	[122]
	Ethosome	Piroxicam	88.8 ± 8.37 nm, zeta potential −11 mV	Iontophoresis significantly enhanced ethosomal piroxicam permeation compared with the free drug	Ex vivo permeation evaluation showed the permeation of ethosomal ones in 1 hour (14.27 ± 2.05%)	Iontophoresis significantly enhanced ethosomal piroxicam permeation and transdermal ethosomal piroxicam along with iontophoresis seems to be promising in wound healing	[123]
	Polymeric nanoparticle	Acidic fibroblast growth factor (aFGF)	297.8 ± 32.17 nm, zeta potential −15.8 ± 1.40 mV	Polymeric nanoparticles enhance skin delivery efficiency of aFGF	Drug exhibited a burst release of ≈1.0 µg/mL (≈40%) from the microneedle in the first four hours	Polymeric nanoparticles MNs achieved a quicker wound closure rate with reduced necrotic tissues, faster re-epithelialization, enhanced collagen deposition, and increased neo-vascularization	[124]
	Polymeric nanoparticle	Dimethyloxalylglycine	125 nm, zeta potential 23.40 ± 2.85	Dimethyloxalylglycine loaded into polymeric nanoparticle can improve the stability of the drug	Polymeric nanoparticles improve skin penetration and retention behavior of Dimethyloxalylglycine	Polymeric nanoparticle arrays would not only exhibit excellent antibacterial activity against pathogenic bacteria but also enhance angiogenesis within wound bed by up-regulating the expression of HIF-1α, leading to a significant therapeutic efficiency on bacteria-infected cutaneous wound healing	[125]
	Transferosome	Deferoxamine (DFO)	109.2 ± 2.04 nm to 265.67 ± 2.41 nm	In vitro study revealed that the DFO-loaded transferosomal gel was found effective in sustaining DFO release	In vitro study revealed that the DFO-loaded transferosomal gel was found effective in sustaining DFO release	The DFO-loaded transferosomal gel increases the rate of neovascularization and increases collagen fiber production	[126]
	Dendrimer	Hesperidin	encapsulation efficiency and drug loading of 20% and 3.33%	Dendritic polymers improve water solubility and bioavailability of drugs	Rat skin treated with hesperidin showed maximum intensity at the skin surface of 0–5 μm, while dendrimer showed a deposition of drug in the epidermis up to 15–25 μm	In vivo results showed that the preparation had better wound contraction activity compared to the control group; after 14 days, the control group had 79 ± 1.41, while the 10% of formulation had 98.9 ± 0.42	[127]
	Inclusion complex	Dihydromyricetin	a spherical shape with cavities or fragments of cavities	Inclusion complex enhances the stability of dihydromyricetin	Inclusion complex exhibited a gradual release in PBS solution at 37 °C, reaching a steady release after approximately 120 min	Inclusion complex significantly reduced the M1 phenotypic transition in RAW264.7 cells, effectively restoring M2 polarization, thereby shortening the inflammatory period.	[128]
Skin Cancer	Liposome	Indocyanine green (ICG)	257 ± 42 nm, zeta potential −65.8 ± 1.9 mV	ICG loaded into liposome can improve the stability of the drug	Skin permeation experiments revealed that the liposomes significantly improved skin permeation of ICG	Liposomes facilitated the cellular uptake, photo-cytotoxicity, and skin permeation of ICG	[129]
	Liposome	Vemurafenib	105.66 ± 12.38 nm, EE% = 98.92 ± 2.36%, zeta potential −4.75 ± 0.86 mV	Liposomes overcome the low solubility of Vemurafenib	In vitro permeation showed that the quantity of Vem penetrating to the receptor of the Franz Diffusion Cell System was significantly higher in the liposome group than Vemurafenib	In vivo experiments confirmed the effective antitumor ability of liposomes delivered via the skin	[130]
	Liposome	ROS-responsive doxorubicin prodrug (pB-DOX), ICG	600 μm in height and 300 μm in diameter	ICG loaded into liposome can improve the stability of the drug	Data showed that after 5 min, the drug content in skin reached 92.20%	The growth of the tumors in the liposome MNs was inhibited by 93.5%, providing a promising candidate for clinical melanoma therapy	[131]
	Polymeric nanoparticle	Polydopamine (PDA)	100 ± 10 nm	Polymeric nanoparticles improve drug bioavailability and skin permeability	Polymeric nanoparticles improve skin penetration and retention behavior of PDA	Polymeric nanoparticle accumulates the high photothermal effect of Cu-PDA NPs (~50.40%) to acquire the energy from NIR irradiation, leading to the generation of a new minimally invasive synergistic therapy.	[132]
	Polymeric micelle	Vismodegib (VSD)	20–30 nm	Polymeric micelles improve Skin Delivery and Biodistribution of Drugs	Application of micelle solution and micelle gel to human skin for 12 h under infinite dose conditions resulted in statistically equivalent VSD deposition (0.62 ± 0.11 and 0.67 ± 0.14 μg/cm^2^, respectively)	Cutaneous delivery of VSD from micelle-based formulations might enable targeted, topical treatment of superficial BCC with minimal risk of systemic exposure	[133]
	Polymeric micelle	TAK-441	10–15 nm	Polymeric micelles improve the solubility of the TAK-441	Finite dose experiments using human skin demonstrated that this formulation resulted in significantly greater cutaneous deposition of TAK-441 after 12 h than a non-micelle control formulation	Polymeric micelles reduce the risk of systemic side effects in vivo for the treatment of basal cell carcinoma	[134]
Others	Zein nanoparticle	Cur	330–400 nm, zeta potential −22 mV	Cur can be incorporated into nanoparticles, which improves the solubility and stability of the drug	Transdermal delivery experiments and porcine skin fluorescence imaging indicated that nanoparticles facilitate the penetration of Cur across the epidermis layer of skin to reach deep-seated sites	Cur-loaded nanoparticles down-regulated the generation of inflammatory cytokines and chemokines in keratinocytes through suppression of the nuclear translocation of NF-κBp65 and hence exerted an anti-dermatitis effect	[135]
	Polymeric nanoparticle	Etoricoxib (ETX)	267.9 ± 9.4 nm, EE% = 95 ± 0.2%, zeta potential 32.9 ± 0.47 mV	Chitosan and hyaluronic acid delivered ETX to the deeper skin layers	Polymeric nanoparticle showed efficient dermal targeting by significantly enhanced percentage of ETX permeated and retained in the various skin layers in comparison to ETX	Polymeric nanoparticle exhibited superior anti-inflammatory properties in vivo compared to ETX in dithranol-induced mice ear dermatitis	[136]
	Polymeric nanoparticle	DOX	129.1 nm, EE% = 23.6%	DOX can be incorporated into nanoparticles, which improves the solubility of the drug	Study shows polymer nanoparticles significantly promote skin penetration	Polymeric nanoparticles facilitated the apoptosis of hypertrophic scar cells, and reduced the burden and progression of hypertrophic scars in a xenografted mouse model	[137]

## Abbreviations

SC	Stratum corneum
NLC	Nanostructured lipid carrier
AmB	Amphotericin B
NC	Nanocrystal
CUR	Curcumin
EA	Edge activator
SLN	Solid lipid nanoparticle
PNP	Polymeric nanoparticle
PM	Polymeric micelle
CMC	Critical micelle concentration
AuNPs	Gold nanoparticles
CD	Cyclodextrins
TRA	Trans retinoic acid
TPP	Tri-phenylphosphine
MTX	Methotrexate
EGCG	Epigallocatechin gallate
NSO	Nigella sativa seed oil
VCO	Virgin coconut oil
PSR	Psoralen
MOP	Methoxypsoralen
aFGF	Acidic fibroblast growth factor
DFO	Deferoxamine
ICG	Indocyanine green
ROS	Reactive oxygen species
PDA	Polydopamine
VSD	Vismodegib
ETX	Etoricoxib
DOX	Doxorubicin
FDA	Food and drug administration
UDL	Ultradeformable liposomes
MelNPs	Melanin-like nanoparticles
UV	Ultraviolet
KGF-2	Keratinocyte growth factor-2
MNs	Microneedles
CSD	Cumulative sun damage
KC	Keratinocyte carcinoma
BCC	Basal cell carcinoma
SCC	Squamous cell carcinoma
AK	Actinic keratoses
WHO	World Health Organization
AD	Atopic dermatitis
HA	Hyaluronic acid
PHA	Poly hyaluronic acid
TIMP-1	Tissue inhibitor of metalloproteinases 1
